# Candidate Vaccine Sequences to Represent Intra- and Inter-Clade HIV-1 Variation

**DOI:** 10.1371/journal.pone.0007388

**Published:** 2009-10-08

**Authors:** Otto O. Yang

**Affiliations:** 1 Division of Infectious Diseases, Department of Medicine, David Geffen School of Medicine, University of California Los Angeles, Los Angeles, California, United States of America; 2 Department of Microbiology, Immunology, and Molecular Genetics, David Geffen School of Medicine, University of California Los Angeles, Los Angeles, California, United States of America; 3 UCLA AIDS Institute, Los Angeles, California, United States of America; New York University School of Medicine, United States of America

## Abstract

A likely key factor in the failure of a HIV-1 vaccine based on cytotoxic T lymphocytes (CTL) is the natural immunodominance of epitopes that fall in variable regions of the proteome, which both increases the chance of epitope sequence mismatch with the incoming challenge strain and replicates the pathogenesis of early CTL failure due to epitope escape mutation during natural infection. To identify potential vaccine sequences to focus the CTL response on highly conserved epitopes, the whole proteomes of HIV-1 clades A1, B, C, and D were assessed for Shannon entropy at each amino acid position. Highly conserved regions in Gag (cGag-1, Gag 148–214, and cGag-2, Gag 253–331), Env (cEnv, Env 521–606), and Nef (cNef, Nef 106–148) were identified across clades. Inter- and intra-clade variability of amino acids within the regions tended to overlap, suggesting that polyvalent representation of consensus sequences for the four clades would allow broad HIV-1 strain representation. These four conserved regions were rich in both known and predicted CTL epitopes presented by a breadth of HLA types, and screening of 54 persons with chronic HIV-1 infection revealed that these regions are commonly immunogenic in the context of natural infection. These data suggest that vaccine delivery of a 16-valent mixture of these regions could focus the CTL response against conserved epitopes that are broadly representative of circulating HIV-1 strains.

## Introduction

Efforts to design a vaccine against Human Immunodeficiency Virus type 1 (HIV-1) have been disappointing. The principal empirical strategies that yielded successful vaccines against other viruses in the past have not provided protective immunity. The first unsuccessful attempts included strategies using inactivated whole virus or virus protein subunits, which would be expected to raise antibodies and HLA class II-restricted helper responses against HIV-1. When such approaches (including a phase III trial of the HIV-1 envelope-based “AIDSVAX”) failed to produce protective humoral immunity, researchers turned to the idea that a vaccine to elicit HLA class I-restricted cytotoxic T lymphocyte (CTL) responses might provide protection against disease if not infection, given the increasingly clearly protective role of CTL in the immunopathogenesis of HIV-1 infection.

The attempts to generate HIV-1-specific CTL responses with a vaccine have focused heavily upon vector development for immunogenicity, as safety concerns precluded the classical empiric approach of using live-attenuated HIV-1. Although numerous strategies ranging from naked plasmid DNA to replication-competent vaccinia vector showed promise in animal models, to date only recombinant adenovirus serotype 5 (rAd5) has appeared to be reliably immunogenic for CTL responses in humans. Vaccination of HIV-1-uninfected persons with modified replication-incompetent rAd5 containing HIV-1 genes elicited CTL responses comparable to those raised by natural HIV-1 infection, as measured by ELISpot and intracellular interferon-γ assays [1]. Unfortunately, the first large efficacy trial of this approach was halted for futility at mid-enrollment, when interim safety analysis revealed that there was no difference in infection incidence or set-point viremia levels after infection between placebo and vaccine arms [2].

The cause of vaccine failure remains unknown, but there are at least two major types of possibilities (reviewed in [3], [4]). First, the “immunogenicity” of the vaccine as reflected by IFN-γ-based assays using exogenously loaded small peptides as surrogates for cellular HIV-1 infection may not have reflected true functional immunogenicity for antiviral CTL, i.e. capability to recognize levels of epitope on infected cells [5] or phenotype/effector/trafficking capability of the CTL raised. While this has yet to be investigated, the ability of the same rAd5 vector to elicit protective immunity against SIV in the rhesus macaque model suggests that immunogenicity from this vector may be adequate. Second, the CTL targeting elicited by the vaccine may have been faulty in at least two ways related to immunodominant targeting of variable epitopes. Recent functional studies of CTL antiviral activity have indicated that the impact of epitope variation has been greatly underestimated by prior peptide-based assays [5], [6]. The targeted epitopes from the vaccine may not have matched the epitopes in the circulating strain in this case. Another possibility is that targeting of variable epitopes may have replicated natural mis-direction of CTL immunodominance. In essence, the vaccine approach in the trial of rAd5 delivered genes for most of the HIV-1 proteome (Gag, Pol, Nef), to mimic natural infection and whole viral immunologic exposure. Presumably, this would give the host similar choices for CTL targeting and result in immunodominance patterns comparable to early natural infection.

While other successful vaccines have targeted viruses where natural immunity is frequently protective if the host survives the initial infection, natural immunity against HIV-1 infection generally fails. Thus, mimicking naturally occurring immune responses with a vaccine may not suffice for HIV-1, in contrast to other viruses. Increasing data about early HIV-1 infection suggest that CTL targeting initially is misdirected towards variable epitopes [7], [8], allowing early viral escape [9], [10], [11], [12], and inadequate early immune containment with resultant irreversible depletion of the CD4^+^ T helper cell pool [13], [14], [15] that dooms the efficacy of the CTL response in the long term despite eventual re-targeting against conserved epitopes. A vaccine might therefore offer the opportunity to alter the patterns of CTL immunodominance seen in natural infection by predetermining memory responses against epitopes that are highly conserved, rather than those with the highest affinity [16] or other immunodominant properties that do not limit escape. Thus, some vaccine developers have considered strategies to provide vaccine sequences to target conserved epitopes, in contrast to the current standard approach of delivering whole proteins (including variable regions). Here we identify conserved regions within the HIV-1 proteome to propose as vaccine sequences, with additional representation of intra- and inter-clade variation.

## Materials and Methods

### Ethics Statement

The portion of this work involving human subjects was performed under a protocol approved by the Office for Protection of Human Research Subjects (IRB) at the University of California Los Angeles. Written informed consent was received from each participant.

### HIV-1 sequences

All available (as of 7/21/08) full length protein sequences of Gag, Pol, Env, Nef, Tat, Rev, Vif, Vpr, and Vpu, for clades A1/A2, B, C, and D were downloaded from the Los Alamos National Laboratory HIV Sequence Database at:http://www.hiv.lanl.gov/components/sequence/HIV/search/search.html.

### Determination of Shannon entropy across the HIV-1 proteome

Shannon entropy is a quantitative measurement of uncertainty in a data set such as a collection of protein sequences. Comparing the sequences against each other, the entropy of each amino acid position reflects the uncertainty (variability) of that position across all sequences. Lower entropy reflects more predictability at that position, e.g. the amino acid is always the same (no entropy), or few different amino acids observed rarely. Higher entropy reflects more uncertainty at that position, e.g. many different amino acids frequently occupy that position with no predominant amino acid.

To calculate Shannon entropy across the HIV-1 proteome, the sequences of each full length protein within each clade were aligned against clade consensus protein sequences (Los Alamos HIV Sequence Database) using Clustal ×2.0.10 on an Apple Macintosh Pro running OS ×10.5.7, with manual editing. Shannon Entropy at each amino acid position was then calculated using the Shannon Entropy online tool at the Los Alamos National Laboratory HIV Sequence Database:http://www.hiv.lanl.gov/content/sequence/ENTROPY/entropy_one.html. (Further explanation is given at the Los Alamos National Laboratory HIV Sequence Database:http://www.hiv.lanl.gov/content/sequence/ENTROPY/entropy_readme.html).

### Listing of known and predicted epitopes within regions of the HIV-1 proteome

Known and predicted CTL epitopes within regions of the HIV-1 proteome were listed using the Epitope Location Finder program online tool at the Los Alamos National Laboratory HIV Immunology Database (using all available class I HLA types):http://www.hiv.lanl.gov/content/sequence/ELF/epitope_analyzer.html.

The input amino acid sequences for epitope prediction were HIV-1 2004 overall consensus sequences (“consensus of consensus”) obtained from the Los Alamos National Laboratory HIV Sequence Database:http://www.hiv.lanl.gov/content/sequence/NEWALIGN/align.html.

### Analysis of amino acid variation within clades

The frequencies of amino acid polymorphisms at specific locations within each clade were assessed using the online QuickAlign tool from the Los Alamos National Laboratory HIV Sequence Database:http://www.hiv.lanl.gov/cgi-bin/QUICK_ALIGN/QuickAlign.cgi.

### HIV-1-infected participants

Persons with chronic HIV-1 infection who were not on antiretroviral therapy were recruited through a University of California Los Angeles IRB-approved protocol. PBMC were isolated from freshly drawn whole blood by ficoll-hypaque gradient and viably cryopreserved. Plasma viremia data were obtained from medical records (or patient self-report in a few cases where medical records were not available).

### Mapping of HIV-1-specific CTL responses

Standard interferon-γ ELISpot assays were performed using polyclonally expanded CD8^+^ T cells as previously described in detail [17]. In brief, PBMC were thawed and cultured with a CD3:CD4 bi-specific monoclonal antibody, resulting in expansion of CD8^+^ T cells (generally at least 95% CD3^+^CD8^+^) that has been shown to mirror CTL responses in unexpanded CD8^+^ T cells [17], [18]. After 14 days, the cells were utilized for standard ELISpot assays using overlapping peptides (15 amino acids sequentially overlapping by 11 amino acids) from the NIH AIDS Reference and Research Reagent Repository. Gag peptides included clade B consensus and DU422 sequences (catalog #8117 and #6869). Env peptides included clade B consensus or MN sequences (#9480 and #6451). Nef peptides included clade B consensus sequences (#5189). Pools of 16 or fewer peptides were utilized for a first round of screening, followed by 4×4 matrices to identify individual peptide candidates for a second round of screening, followed by confirmation of individual peptides in a third round of screening.

## Results

### Despite the overall variability of the HIV-1 clade B proteome, there are stretches of relatively conserved sequences

**Figure 1:**
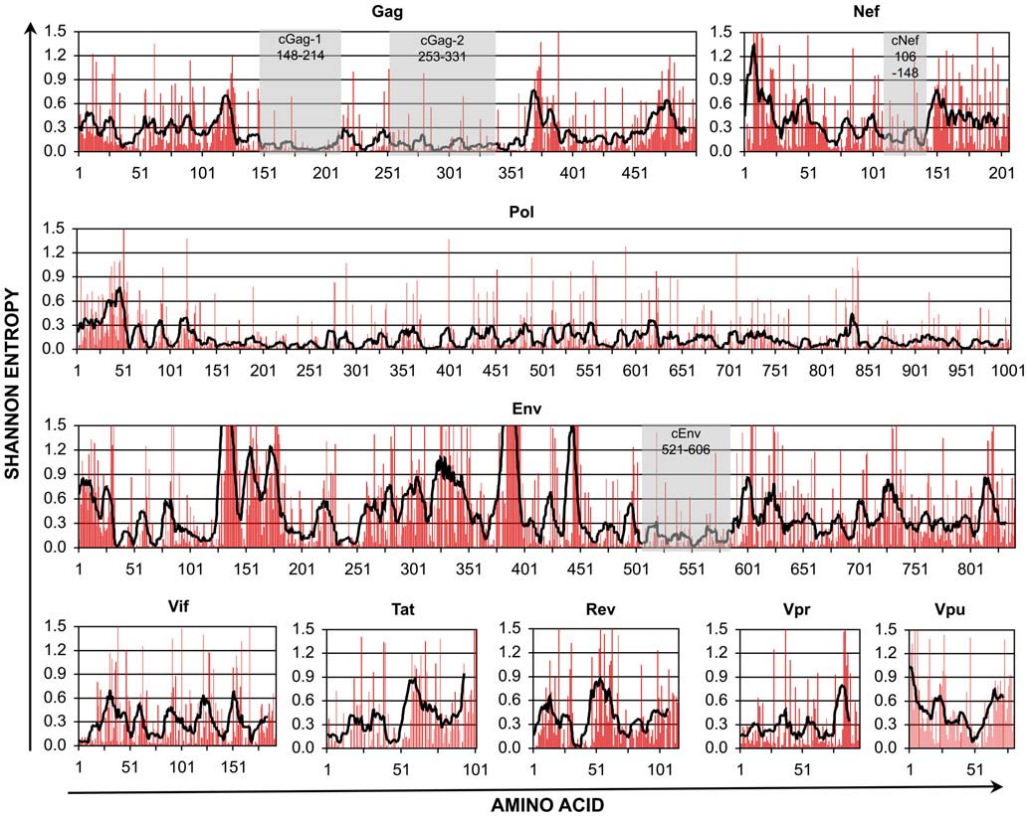
Shannon Entropy across the HIV-1 clade B proteome. All full length clade B protein sequences in the LANL HIV Sequence Database were aligned and assessed for Shannon entropy of each amino acid position. The red bars indicate entropy at each codon, and the heavy black lines plot mean entropy for the nine amino acids starting at each position. The shaded regions indicate relatively conserved regions that were examined further. Note that the numbering of the conserved regions (based on HXB2 position) does not necessarily match amino acid positions in the graphs (due to insertions in some sequences relative to HXB2).

The plasticity of HIV-1 sequences is an obvious barrier for vaccine development. To assess sequence variability and conservation across the viral proteome, all available clade B complete HIV-1 protein sequences in the Los Alamos National Laboratory (LANL) HIV Sequence Database were assessed for Shannon entropy at each amino acid position. The entropy at each position and the mean entropy for each stretch of nine amino acids (corresponding to most potential epitopes) were plotted for each of the nine viral proteins (Figure 1). The plot revealed gross differences between proteins, such as generally higher variability in Env and relatively lower variability in Pol. All proteins contained spikes of variable codons scattered throughout, although there were stretches of lower variability with fewer spikes even in the generally more variable proteins Env and Nef. These plots therefore demonstrated that despite the overall plasticity of the HIV-1 proteome, there are sequence constraints that constrict variability in certain regions within several proteins. Four regions from highly expressed proteins (i.e. excluding Pol) that were at least 40 amino acids in length and generally conserved were selected for further examination (Figure 1 shaded regions). Pol was excluded due to its low level of expression from translation requiring a ribosomal frameshift along the *gag-pol* transcript [19], which can reduce the immunogenicity and antiviral efficacy of Pol-specific CTL [20], [21].

### Sequence constraints fall in the same regions across different HIV-1 clades, and within selected conserved regions, inter-clade variability is limited to consistent sites

**Figure 2:**
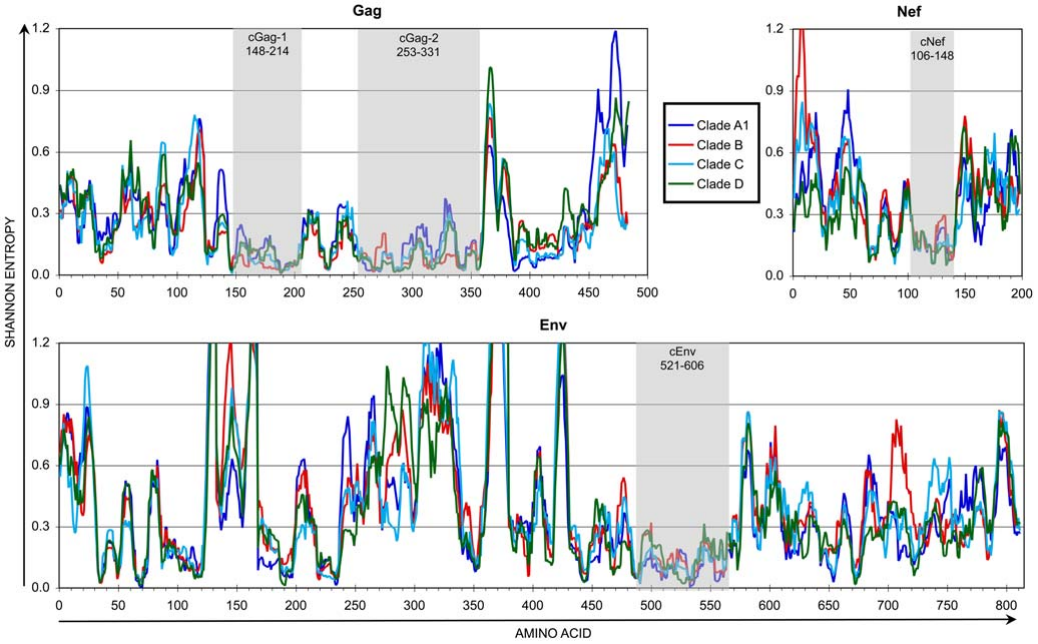
Entropy of 9 amino acid stretches for clades A1-D of HIV-1. Shannon entropy was assessed for full length protein sequences of clades A1, C, and D available from the LANL HIV Sequence Database, as in Figure 1. For each clade, the mean entropies of nine amino acid stretches are plotted for each clade for the proteins containing the relatively conserved regions (shaded) identified in Figure 1. Note that the numbering of the conserved regions (based on HXB2 position) does not necessarily match amino acid positions in the graphs (due to insertions in some sequences relative to HXB2).

**Figure 3:**
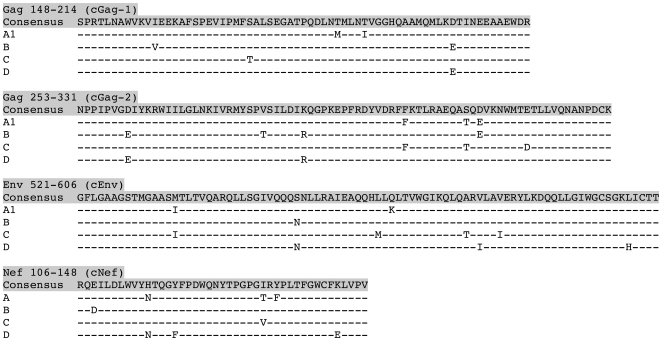
Sequences of relatively conserved regions of the HIV-1 proteome. The relatively conserved regions shaded in Figures 1 and 2 are given for clades A1, B, C, and D, aligned against the overall consensus across all group M clades. “-” indicates amino acid identity with overall consensus.

HIV-1 is observed in genetically distinct clades that reflect divergent pathways for evolution [22]; clades A (A1), B, C, and D reflect the vast majority of circulating strains worldwide. To assess whether the patterns of sequence variability observed for clade B (Figure 1) apply to other clades, the entropy for available clade A1, C, and D HIV-1 complete protein sequences in the LANL HIV Sequence Database were analyzed in parallel for Shannon entropy at each amino acid position. Comparisons of the clades revealed that the patterns of variability across the proteome were very similar in general (Figure 2 and data not shown), suggesting that different HIV-1 clades share certain areas of functional/structural constraint despite divergent evolution. Examining the four selected conserved regions, it was notable that differences between clade consensus sequences in these regions were limited to a few codon positions (Figure 3). This finding further suggested that inter-clade variability in these conserved regions is relatively limited, with a few wobble positions that have diverged between clades.

### Within conserved regions of the HIV-1 proteome, much of the intra-clade diversity overlaps inter-clade sequence variation

**Figure 4:**
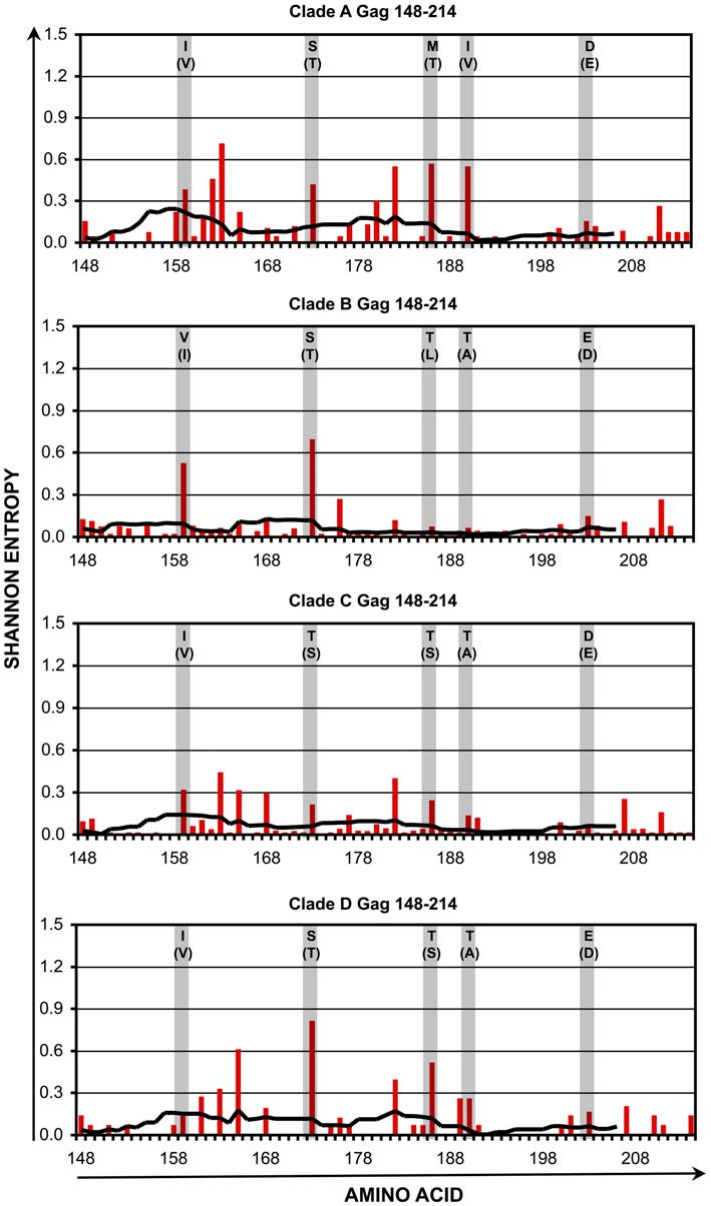
Shannon entropy in the conserved region cGag-1. For HIV-1 Gag amino acids 148–214, Shannon entropies of individual codons (red bars) and average entropies of stretches of 9 codons (black lines) are plotted for clades A1–D. The shaded columns indicate positions where the consensus sequences differ between clades. For each of those positions, the consensus amino acid and most common variant (in parentheses) are indicated.

**Figure 5 pone-0007388-g005:**
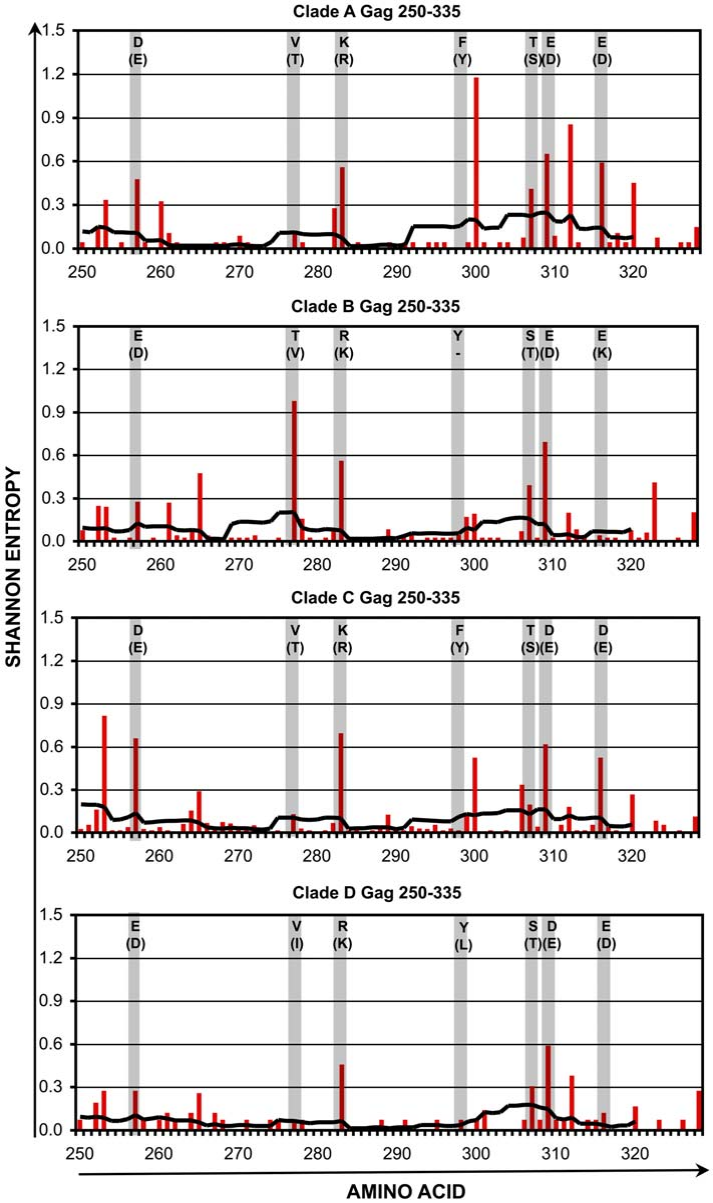
Shannon entropy in the conserved region cGag-2. For HIV-1 Gag amino acids 250–335, Shannon entropies of individual codons (red bars) and average entropies of stretches of 9 codons (black lines) are plotted for clades A1–D. The shaded columns indicate positions where the consensus sequences differ between clades. For each of those positions, the consensus amino acid and most common variant (in parentheses) are indicated.

**Figure 6 pone-0007388-g006:**
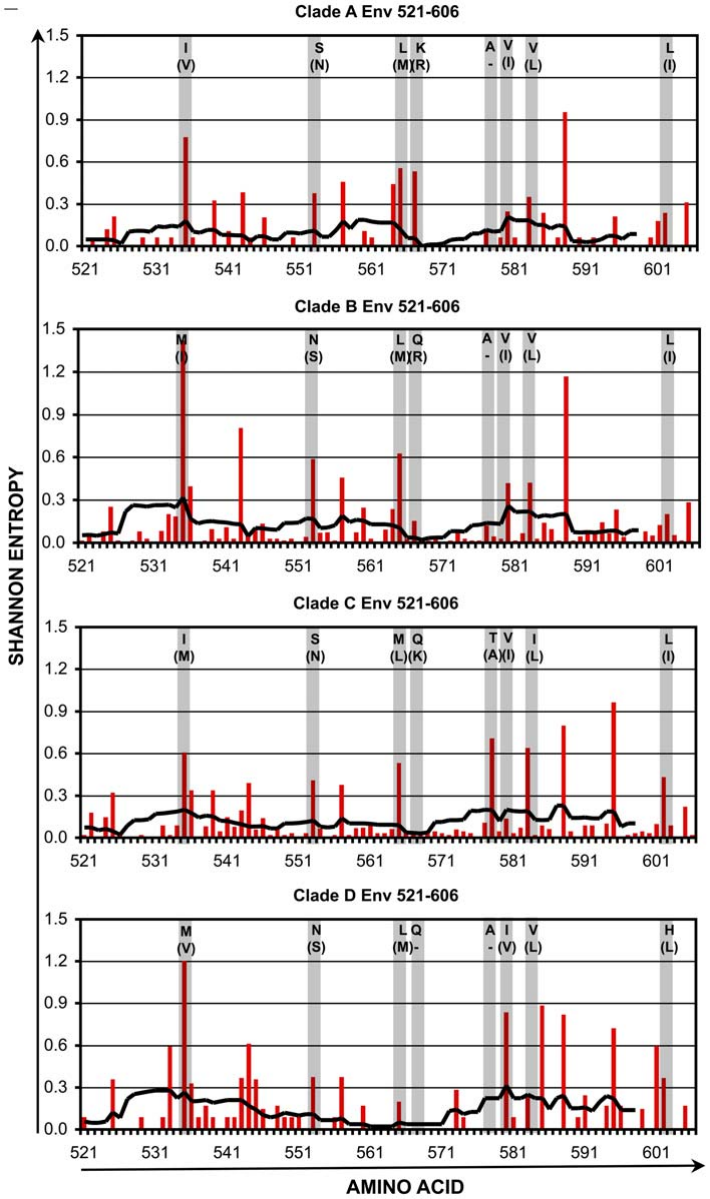
Shannon entropy in the conserved region cEnv. For HIV-1 Env amino acids 521–606, Shannon entropies of individual codons (red bars) and average entropies of stretches of 9 codons (black lines) are plotted for clades A1–D. The shaded columns indicate positions where the consensus sequences differ between clades. For each of those positions, the consensus amino acid and most common variant (in parentheses) are indicated.

**Figure 7:**
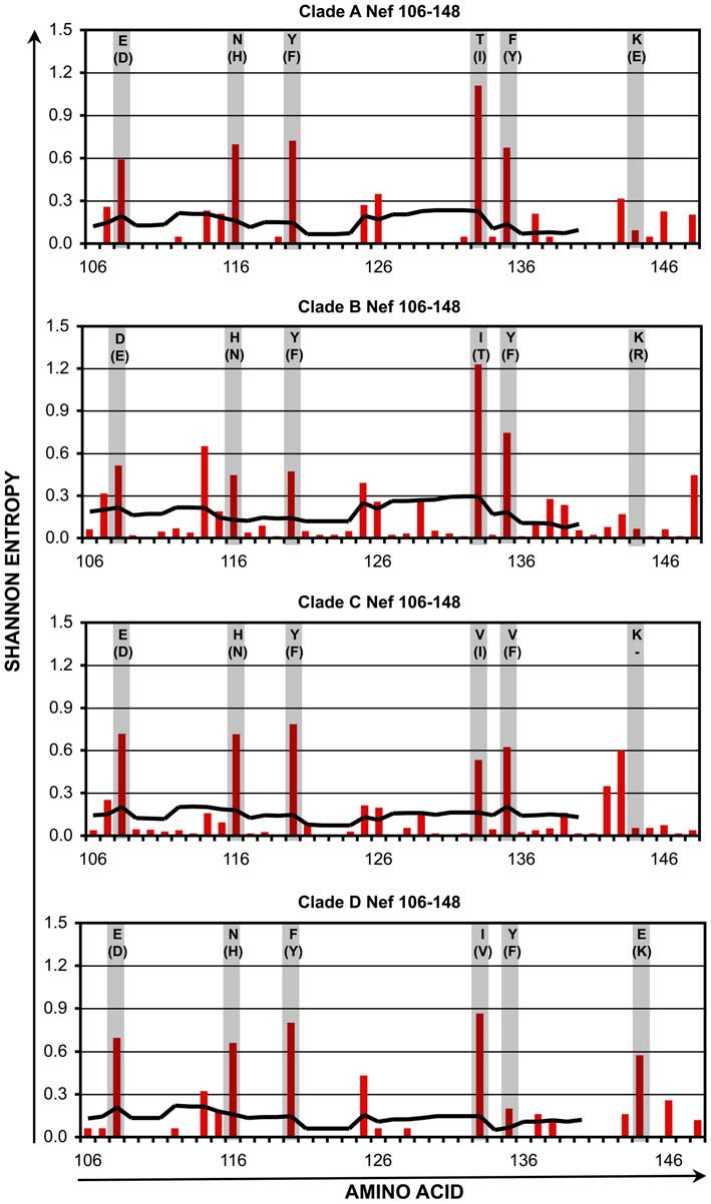
Shannon entropy in the conserved region cNef. For HIV-1 Nef amino acids 106–148, Shannon entropies of individual codons (red bars) and average entropies of stretches of 9 codons (black lines) are plotted for clades A1–D. The shaded columns indicate positions where the consensus sequences differ between clades. For each of those positions, the consensus amino acid and most common variant (in parentheses) are indicated.

Within the four selected conserved regions, there remained amino acid positions that are somewhat variable within each clade. The variability in these regions was assessed in further detail for clades A1-D (Figures 4, 5, 6, 7 and Tables 1, 2, 3, 4). Plotting the entropy of each amino acid position (Figures 4–7) within each clade demonstrated that many of the positions that were more variable intra-clade overlapped positions where the consensus sequence differed inter-clade. Furthermore, at these varying positions within each clade, there often was a single dominant non-consensus polymorphism that corresponded to the consensus amino acid for another clade. For example, in region cGag-1, the consensus amino acid at position 159 was isoleucine for clades A1, C, and D, and valine for clade B. Within clades A1, C, and D, position 159 often varied from consensus, and the most common polymorphism was valine. Conversely for clade B, position 159 also frequently varied from consensus, and the most common polymorphism was isoleucine. These observations suggested that there is a high degree of overlap between intra-and inter- clade variability in these conserved regions, consistent with a recent study examining HIV-1 sequence variation [23]. Thus a mixture of clade consensus sequences of these conserved regions (to represent HIV-1 variability worldwide) would represent a large portion of circulating HIV-1 sequences within any clade (to represent HIV-1 variability within individuals).

**Table 1:** Varying consensus sequences and common polymorphisms in cGag-1.

		159	173	186	190	203
**CLADE A1**	Consensus	**I (90.4%)**	**S (91.2%)**	**M (85.5%)**	**I (87.2%)**	**D (95.2%)**
	Polymorphism #1	V (9.6%)	T (7.2%)	T (8.9%)	V (8.8%)	E (4.8%)
	Other				T (2.4%)	
**CLADE B**	Consensus	**V (79.4%)**	**S (84.5%)**	**T (99.1%)**	**T (99.4%)**	**E (96.5%)**
	Polymorphism #1	I (20.5%)	T (14.8%)	L (0.4%)	A (0.3%)	D (3.5%)
**CLADE C**	Consensus	**I (90.7%)**	**T (95.6%)**	**T (95.6%)**	**T (97.8%)**	**D (99.0%)**
	Polymorphism #1	V (9.1%)	S (3.4%)	S (3.2%)	A (1.4%)	E (1.0%)
**CLADE D**	Consensus	**I (96.2%)**	**S (65.8%)**	**T (88.5%)**	**T (94.9%)**	**E (96.2%)**
	Polymorphism #1	V (3.8%)	T (30.4%)	S (6.4%)	A (2.5%)	D (3.8%)

At each position in cGag-1 (Gag 148–214) where consensus amino acids differ between clades, the frequency of the consensus amino acid and the most common polymorphism(s) within each clade are listed (based on all available whole protein sequences in the LANL HIV Sequence Database).

**Table 2:** Varying consensus sequences and common polymorphisms in cGag-2.

		260	280	286	301	310	312	319
**CLADE A1**	Consensus	**D (88.7%)**	**V (97.6%)**	**K (78.4%)**	**F (99.2%)**	**T (87.1%)**	**E (68.0%)**	**E (79.8%)**
	Polymorphism #1	E (10.5%)	T (1.6%)	R (21.0%)	Y (0.8%)	S (12.9%)	D (32.3%)	D (19.4%)
**CLADE B**	Consensus	**E (92.4%)**	**T (73.0%)**	**R (75.3%)**	**Y (99.7%)**	**S (91.0%)**	**E (69.5%)**	**E (99.7%)**
	Polymorphism #1	D (7.4%)	V (18.0%)	K (24.7%)	-	T (8.9%)	D (30.5%)	K (0.2%)
	Other		S (3.6%) I (3.4%)					
**CLADE C**	Consensus	**D (68.5%)**	**V (97.5%)**	**K (60.1%)**	**F (99.8%)**	**T (96.6%)**	**D (70.1%)**	**D (80.5%)**
	Polymorphism #1	E (31.0%)	T (1.7%)	R (39.6%)	Y (0.2%)	S (3.1%)	E (29.6%)	E (18.8%)
**CLADE D**	Consensus	**E (91.1%)**	**V (98.7%)**	**R (83.5%)**	**Y (97.5%)**	**S (91.1%)**	**D (72.2%)**	**E (97.5%)**
	Polymorphism #1	D (7.6%)	I (1.3%)	K (16.5%)	L (1.3%)	T (8.9%)	E (26.6%)	D (2.5%)

At each position in cGag-2 (Gag 250–335) where consensus amino acids differ between clades, the frequency of the consensus amino acid and the most common polymorphism(s) within each clade are listed (based on all available whole protein sequences in the LANL HIV Sequence Database).

**Table 3:** Varying consensus sequences and common polymorphisms in cEnv.

		500	518	530	532	543	545	548	567
**CLADE A1**	Consensus	**I (73.2%)**	**S (83.1%)**	**L (88.7%)**	**K (82.9%)**	**A (100%)**	**V (95.8%)**	**V (90.1%)**	**L (97.2%)**
	Polymorphism #1	V (14.1%)	N (15.5%)	M (9.9%)	R (15.5%)	-	I (2.8%)	L (7.0%)	I (2.8%)
	Other	M (7.0%)							
**CLADE B**	Consensus	**M (27.8%)**	**N (84.2%)**	**L (73.7%)**	**Q (97.4%)**	**A (99.7%)**	**V (89.8%)**	**V (91.0%)**	**L (96.4%)**
	Polymorphism #1	I (26.3%)	S (10.5%)	M (24.7%)	R (1.4%)	-	I (7.4%)	L (3.8%)	I (1.5%)
	Other	V (23.7%	R (4.8%)	I (1.2%)	K (0.9%)		L (2.8%)	I (3.6%)	
**CLADE C**	Consensus	**I (85.5%)**	**S (86.1%)**	**M (79.8%)**	**Q (99.6%)**	**T (45.9%)**	**V (97.5%)**	**I (82.9%)**	**L (91.2%)**
	Polymorphism #1	M (7.3%)	N (13.3%)	L (19.8%)	K (0.4%)	A (53.9%)	I (2.2%)	L (8.4%)	I (3.3%)
	Other	V (4.3%)							V (2.2%)
**CLADE D**	Consensus	**M (15.3%)**	**N (91.8%)**	**L (94.1%)**	**Q (100%)**	**A (100%)**	**I (40.5%)**	**V (94.1%)**	**H (90.6%)**
	Polymorphism #1	L (57.6%)	S (4.7%)	M (5.9%)	-	-	V (57.1%)	L (3.5%)	L (7.1%)
	Other	V (17.6%) I (8.2%)							

At each position in cEnv (Env 521–606) where consensus amino acids differ between clades, the frequency of the consensus amino acid and the most common polymorphism(s) within each clade are listed (based on all available whole protein sequences in the LANL HIV Sequence Database).

**Table 4:** Varying consensus sequences and common polymorphisms in cNef.

		108	116	120	133	135	144
**CLADE A1**	Consensus	**E (75.0%)**	**N (50.7%)**	**Y (63.7%)**	**T (40.4%)**	**F (40.3%)**	**K (98.5%)**
	Polymorphism #1	D (24.3%)	H (49.3%)	F (34.1%)	I (39.0%)	Y (59.7%)	E (1.5%)
	Other				V (18.4%)		
**CLADE B**	Consensus	**D (83.0%)**	**H (84.7%)**	**Y (84.6%)**	**I (52.4%)**	**Y (70.1%)**	**K (99.3%)**
	Polymorphism #1	E (16.5%)	N (15.1%)	F (15.0%)	T (22.8%)	F (27.4%)	R (0.3%)
	Other				V (22.3%)	W (1.5%)	
**CLADE C**	Consensus	**E (76.9%)**	**H (62.1%)**	**Y (44.0%)**	**V (85.8%)**	**Y (81.4%)**	**K (99.5%)**
	Polymorphism #1	D (22.7%)	N (37.4%)	F (54.7%)	I (8.1%)	F (12.9%)	-
**CLADE D**	Consensus	**E (53.5%)**	**N (35.4%)**	**F (30.3%)**	**I (72.4%)**	**Y (96.0%)**	**E (83.7%)**
	Polymorphism #1	D (46.5%)	H (64.6%)	Y (64.6%)	V (13.3%)	F (3.0%)	K (9.2%)
	Other			I (5.1%)	T (12.2%)		Q (7.1%)

At each position in cNef (Nef 106–148) where consensus amino acids differ between clades, the frequency of the consensus amino acid and the most common polymorphism(s) within each clade are listed (based on all available whole protein sequences in the LANL HIV Sequence Database).

### The selected conserved regions are rich in potential CTL epitopes

To assess whether these regions might be immunogenic if delivered in a CTL-based vaccine, the Los Alamos HIV Immunology Database was scanned for known and predicted CTL epitopes falling within the regions (Table 5). Each region contained many previously reported CTL epitopes associated with a variety of HLA types, as well as binding motifs for many additional HLA types. These findings suggested that these conserved regions contain many epitopes that can be presented by a broad range of HLA types, and therefore should be immunogenic for CTL responses from most persons.

**Table 5:** Known and potential epitopes in the conserved regions.

Region	Reported Epitopes (LANL Database)	Potential Epitopes For Other HLA Types
**cGag-1**	A*02, A*11, A*25, A*26	A*03, A*20, A*30, A*31, A*32, A*33, A*68, A*69
	B*04, B*07, B*14, B*15, B*27, B*35, B*38, B*39, B*40, B*42, B*44, B*45, B*52, B*53, B*81	B*08, B*18, B*37, B*46, B*51, B*54, B*55, B*56, B*67, B*78
	C*01, C*06, C*08	C*03, C*04, C*06, C*07
**cGag-2**	A*02, A*11, A*24, A*26, A*33	A*03, A*25, A*29, A*30, A*31, A*66, A*68, A*69
	B*07, B*08, B*14, B*15, B*18, B*27, B*35, B*44, B*52, B*53, B*57, B*58, B*70, B*71, B*81	B*37, B*38, B*39, B*40, B*46, B*48, B*51, B*54, B*55, B*56, B*67, B*78
	C*03, C*04, C*05, C*08, C*18	C*06, C*07, C*12, C*14, C*16
**cEnv**	A*02, A*11, A*23, A*24	A*03, A*25, A*26, A*29, A*30, A*31, A*33, A*66, A*68, A*69
	B*08, B*14, B*27, B*51, B*58	B*07, B*15, B*18, B*35, B*37, B*38, B*39, B*46, B*48, B*52, B*78
	C*03, C*07, C*08, C*12, C*15	C*01, C*02, C*05, C*06, C*16, C*17, C*18
**cNef**	A*01, A*02, A*03, A*23, A*24, A*29, A*33, A*69	A*11, A*25, A*26, A*30, A*31, A*66, A*68
	B*07, B*13, B*15, B*18, B*27, B*35, B*37, B*42, B*49, B*53, B*57	B*38, B*39, B*40, B*44, B*46, B*51, B*54, B*55, B*56, B*58, B*67, B*78
	C*04, C*06, C*07	C*03, C*14

For each region, HLA types for which there are reported and predicted epitopes in the Los Alamos HIV Immunology Database (http://www.hiv.lanl.gov/content/sequence/ELF/epitope_analyzer.html) are listed. Types in bold font are those in the database that have been reviewed as best defined optimal epitopes [42].

### The selected conserved regions are immunogenic in natural infection

To assess this prediction and test whether the selected conserved regions are indeed commonly immunogenic in actual HIV-1 infection, the CTL responses within 54 persons in the Los Angeles area with chronic infection (not receiving antiretroviral treatment) were examined. HIV-1-specific CTL responses were defined by standard interferon-γ ELISpot assays using proteome-spanning 15-mer peptides overlapping by 11 amino acids. All four regions were targeted by CTL within multiple persons (Table 6). The least often recognized was the Env region, where responses were seen in 4/54 persons (7.4%). The two Gag and Nef regions were each targeted similarly in frequency, at 17/54 (31.5%), 17/54 (31.5%), and 19/54 (35.2%) respectively. Across the group, 38/54 (70.4%) of persons targeted at least one region, 17/54 (31.5%) targeted at least two regions, 2/54 (3.7%) targeted at least three regions, and none was observed to target all four regions. Although these subjects were enriched for HLA B*57 due to the bias of selecting untreated persons, the presence or absence of B*57 did not appear to predict responsiveness against these regions. Correlations between targeting of these conserved regions to viremia were not observed in this relatively small number of subjects (not shown). Overall, these data confirmed that these conserved regions can be immunogenic in the context of whole HIV-1 infection, suggesting that CTL responses could be focused preferentially on these regions by an exclusive vaccine.

**Table 6:** Recognition of conserved regions by CTL responses in persons with chronic HIV-1 infection.

ID	VL	HLA A	HLA B	HLA C	cGag1	cGag2	cEnv	cNef	Min# Epitopes
1	Y	*02/*03	*07/*35	*04/*07					0
5	Y	*02/*24	*44/*55	*03/*05		6820/6821			1
6	Y	*01/*02	*08/*35	*04/*07		6811			1
7	N	*29/*68	*44/*53	*04/*16	6791/6797	6823		5171/5172	4
9	Y	*03/*26	*15/*38	*03/*12	6784/6788/7912/7913 7920	6813/6814/6820/6821 7938/7939/7944/7945	8900/8901		4
10	N	*01/*25	*18/*57	*06/*12	6787/6788/6789/6797			5167	4
11	Y	*01/*11	*35/*57	*04/*06					0
12	N	*30/*34	*53/*57	*03/*08	6787/6797			5167/5168	3
14	Y	*03/*25	*18/*57	*07/*12	6787				1
15	Y	*02/*03	*15/*56	*01/*03					0
16	Y	*03/*32	*18/*40	*02/*07					0
17	Y	*02/*03	*44/*51	*04/*14	6787/7911/7912			5168	2
18	Y	*03/*68	*55/*57	03/*06	6797			5167	2
19	Y	*02	*44/*50	*06/*16					0
21	Y	*02/*36	*53	*04				5171/5172	1
22	Y	*02/*32	*07/*15	*03/*07				5172	1
23	Y	*03/*33	*44/*57	*03/*08					0
24	N	*02	*13/*15	*06/*07			6349		1
25	Y	*02/*03	*15/*40	*02/*03		6813/6814		5168	2
26	Y	*01/*02	*08/*44	*05/*07		6811/6814/6815/7940			2
27	Y	*03/*66	*35/*49	*04/*07					0
28	Y	*03/*33	*07/*65	*07/*08		7946	8900		2
29	N	*29/*31	*48/*56	*01/*08	7919/7920				1
30	Y	*30	*08/*81	*07/*18		6812		5167/5168/5170/5171	3
31	Y	*02	*13/*15	*03/*06					0
32	Y	*03/*68	*08/*57	*06/*07	7911/7912				1
33	Y	*01/*74	*49/*53	*04/*07				5171/5172	1
34	Y	*11/*30	*52/*57	*07/*12	6787	6823/6824/7949/7950			2
35	Y	*01/*23	*07/*44	*04/*07				5169/5171	2
36	Y	*02/*11	*39/*57	*06/*07					0
37	Y	*25/*33	*14/*38	*08/*12					0
39	Y	*31/*32	*12/*35	*04/*06					0
40	Y	*02/*31	*35/*44	*04/*05				5169	1
41	Y	*11/*31	*13/*52	*04/*12					0
44	Y	*02/*11	*07/*15	*03/*07		7938/7939			1
45	Y	*30/*68	*15	*02/*03		6820			1
46	N	*25/*68	*35/*14	*04/*08	6792/7915/7916			5168/5169/5171/5172	3
47	Y	*11/*33	*55/*78	*03/*16					0
48	N	*24/*30	*15/*27	*01/*02		6812/6813			1
49	Y	*02/*26	*38/*44	*07/*12	7919/7920				1
50	Y	*24/*30	*38/*44	*05/*12			8907/8908	5171/5172	2
51	Y	*02/*24	*14/*49	*07/*08		7944/7945/7946		5167/5169	4
52	Y	*01/*25	*18/*57	*06/*12	6797/7911/7912			5171	3
54	Y	*29/*74	*50/*81	*06/*18	6793/7915/7916	6814/6815			2
58	N	*02/*29	*44/*15	*03					0
59	Y	*02/*26	*44/*52	*03/*04	7912/7913				1
60	N	*30	*07/*57	*15/*18					0
65	N	*34/*74	*53/*57	*04/*07	6787/6788/7911/7912			5171/5172	2
66	Y	*02/*23	*07/*45	*07/*16				5166/5168/5170/5172	4
67	Y	*11/*25	*18/*27	*01/*12		6813/7936/7937			3
68	Y	*01/*29	*44/*57	*05/*06	6787/7912			5167/5168	2
69	Y	*02	*44	*05/*07		7948			1
70	Y	*02/*74	*44/*53	*04/*05		6823/6824/7947			1
71	N	*03/*33	*53/*57	*04/*18					0
**TOTAL PERSONS RESPONDING (%):**					17 (31.5%)	17 (31.5%)	4 (7.4%)	19 (35.2%)	

A panel of 54 persons (from the Los Angeles area) with chronic HIV-1 infection and not receiving antiretroviral treatment was screened for HIV-1-specific CTL responses by IFN-γ ELISpot assays using overlapping peptides spanning each protein. Gag-specific responses were screened using peptides based on clade B consensus and strain DU422 sequences. Env-specific responses were screened using peptides based on clade B consensus or strain MN sequences. Nef-specific responses were screened using peptides based on clade B consensus sequence. The presence or absence of detectable viremia is indicated in the second column; these individuals were biased towards slow progressors due to recruitment selection for being untreated. Recognized peptides that fall entirely within each conserved region are indicated by NIH AIDS Reagent Repository catalog number. The minimal number of epitopes recognized by each person is listed in the last column (assuming that consecutive overlapping peptides contain a single epitope).

## Discussion

While a CTL based vaccine may not prevent HIV-1 infection, it may offer the opportunity to attenuate disease from subsequent infection. To do so, however, the CTL responses generated by the vaccine must surpass the efficacy of those that would be raised in the natural course of infection. Simply priming CTL responses to have a head start versus HIV-1 infection and immune damage could be advantageous, but the recent failure of an apparently immunogenic CTL-based vaccine to have any impact on viremia set-point [2] suggests that this is not sufficient. Clearly, the interaction between CTL and HIV-1 during acute HIV-1 infection is crucial, because set-point viremia (which predicts the long term rate of disease progression) is determined by the end of acute infection, and is maintained in large part by the CTL response [24], [25], [26]. Given the tendency of this response to target variable epitopes and the observation of rapid and frequent viral escape during acute infection (when massive depletion of crucial helper CD4^+^ T cells occurs), use of a vaccine to focus the response selectively against highly conserved epitopes may offer an avenue to address a key shortcoming of the natural immune response. The other key advantage of such targeting would be minimizing the chance that the epitope sequences of the vaccine would mis-match those of an incoming challenge strain of HIV-1.

Despite the overall plasticity of the HIV-1 proteome, viral variability is not limitless. Its protein sequences clearly are constrained in particular domains, which is not surprising given the many structure/function requirements for viral replication that are imposed on the small proteome. Shannon entropy analysis reveals stretches that are apparently highly constrained in variability and therefore may be useful for CTL focusing by a vaccine. While these regions were selected purely on the basis of entropy analysis, it is interesting that they closely correspond to distinct functional domains in Gag, Env, and Nef. The cGag-1 and cGag-2 sequences both fall in the p24 capsid; the former spans four α-helices that are absolutely required for viability, and the latter spans the so-called “major homology region” that is structurally conserved across other diverse lentiviruses such Feline Immunodeficiency Virus, Rous Sarcoma Virus, Murine Mammary Tumor Virus, Bovine Leukemia Virus, Friend Murine Leukemia Virus, and HTLV-1 [27]. The cEnv region falls in the ectodomain of gp41 and spans the functionally crucial fusion peptide and hepatad repeats [28]. The cNef region falls in the “central conserved region” and includes the second alpha helix (key for interaction with the *src* kinase SH3) and the three adjacent beta strands that compose the central structured core domain [29], and thus contains a key binding site required for many cellular effects of Nef, and sequences that are a crucial structural scaffold.

Within these generally conserved regions, the remaining variability tends to occur at a few amino acid positions, further reflecting the strict functional restrictions for sequence variation. Much of the intra-clade variability overlaps that of the inter-clade variability of these sequences, suggesting that there are limited choices for amino acid substitutions in a few foci of “wobble” within the context of locally rigid functional/structural constraints. Thus, designing a vaccine to represent the polymorphisms seen in the most common clades of HIV-1 serves two advantages of allowing the vaccine to represent the variability between viral species across geographic regions (different clades) and within individuals (genetic drift).

These conserved regions therefore could be utilized in a polyvalent vaccine. A mixture of clades A1-D consensus sequences for each of the four regions would provide highly conserved epitopes, and include the most common variants of epitopes spanning the few “wobble” positions that vary intra- and inter-clade. Delivering these regions separately (in a mixture of 16 individual vectors) rather than concatenating them into a single construct, could be advantageous for preventing competition between epitopes and yield a broader response [30], [31], [32]. While providing the four consensus versions of each region may help represent the breadth of polymorphisms in these overall conserved sequences, another benefit may be dilution of less common variants. An epitope sequence that is conserved in all four consensus sequences will be expressed at four-fold the level of an epitope variant that is present in only one consensus sequence. Although such a polyvalent approach may be cumbersome logistically, there is ample precedent for polyvalent vaccines against other pathogens such as *Streptococcus pneumoniae* [33] and Human Papilloma virus [34].

Other groups also have devised differing strategies for including highly conserved epitopes in CTL-based vaccines. Rolland *et al* identified stretches of 8 or more amino acids selected to be highly conserved across the entire M-group for use as a peptide-based vaccine [35]. This approach identified 46 peptides (listed as “first tier” peptides in: http://www.mullinslab.microbiol.washington.edu/HIV/Rolland2007/Rolland2007-supplement_files/SuppFigs.html#S1), but most of these do not correspond to known epitopes, and ten consist of eight amino acid sequences (unlikely to contain epitopes, which are usually nine amino acids). Thus, this approach may be too stringent to provide the breadth of epitopes required to cover a breadth of HLA types, and additionally faces the difficulty that peptide-based vaccines generally have had poor immunogenicity in humans. More similar to our proposed strategy, Letourneau *et al* [36] screened for stretches of conserved sequences, identifying 14 candidate proteome regions that are relatively conserved. As a vaccine candidate, they concatenated these into a monovalent linear genetic construct, and alternated clade A–D consensus sequences for each consecutive region in an attempt to minimize clade bias. In contrast to our proposed polyvalent vaccine, this approach may not adequately account for intra- and inter-clade polymorphisms within the conserved sequences, because each stretch represents the sequence of only one clade. Concatenation of the 14 regions also could yield unwanted spurious epitopes at the 13 sites of splicing, although testing of this strategy in transgenic human A*02 mice suggested that it can be immunogenic for proper A*02-restricted CTL responses.

The utility of our proposed strategy depends on the hypothesis that vaccine delivery of the selected sequences will steer the early CTL response towards highly constrained epitopes that do not allow escape. While the determinants of effective CTL targeting remain hypothetical, increasing data suggest that highly conserved epitopes in highly expressed proteins are advantageous. Across large numbers of persons, targeting of Gag, a highly expressed and relatively conserved protein that should therefore yield epitopes that are highly expressed and conserved compared to epitopes from other proteins *on average*, has been shown to trend with better immune control [37], [38], [39], [40]. Additional studies on cohorts large enough to power statistical dissections of individual epitope contributions (rather than protein targeting in general) to set-point viremia have begun to suggest reveal epitopes that are associated with better immune containment. Recent data [41] correlated CTL targeting against six epitopes to a beneficial effect in lowering viremia in a cohort of chronically-infected persons. Interestingly, when results of the STEP trial were analyzed, vaccine-induced targeting of any of these epitopes also was associated with lower viremia set-point in persons who had subsequent HIV-1 infection. Of note, four of these six epitopes fall in our proposed vaccine regions (which were designed before these associations were reported): two in cGag-2 (KRWIILGLNK, Gag 263–272, and DRFFKTLRA, Gag 298–306) and two in cNef (HTQGYFPDW, Nef 116–124, and LTFGWCFKLV, Nef 137–146). These data suggest that our sequences have been selected based on properties that yield advantageous epitopes (although all advantageous epitopes do not necessarily fall in these regions).

The optimal vector system and viral sequences to be delivered remain to be determined in HIV-1 vaccine development. Our described sequences are proposed for the latter, given the high degree of sequence conservation and immunogenicity in natural HIV-1 infection. About 70% of persons had detectable responses against at least one of the four regions, suggesting that a sizeable proportion of the general population have HLA types that would allow immunogenicity. A vaccine containing only these regions could have a higher response rate, because it is likely that in natural infection, potentially recognized epitope responses against these regions are masked by the immunodominance of other epitopes outside these regions, and possibly limited by “original antigenic sin.” It is also unclear whether the four chosen regions are equally immunogenic; only about 7% of persons with chronic infection responded against cEnv, while cGag-1, cGag-2, and cNef had similar response rates of about 32 to 35%. This agreed with the lesser number of previously reported epitopes in cEnv compared to the other regions. The number of predicted epitopes in cEnv was similar, however, suggesting that there might be some intrinsic reason for reduced immunogenicity of Env due to some factor such as protein trafficking and accessibility to the class I antigen pathway.

In conclusion, we have identified highly conserved regions of the HIV-1 proteome that exhibit few variable amino acids. These regions appear to be reasonably immunogenic in natural infection. Representation of inter-clade variability within these regions allows coverage of much of the intra-clade variability at those positions. These data support the consideration of polyvalent mixtures of these sequences as vaccine inserts to pre-set memory CTL responses against highly conserved epitopes, thereby favorably altering the immunodominance pattern in subsequent natural infection.

## References

[1] McElrath MJ, De Rosa SC, Moodie Z, Dubey S, Kierstead L (2008). HIV-1 vaccine-induced immunity in the test-of-concept Step Study: a case-cohort analysis.. Lancet.

[2] Buchbinder SP, Mehrotra DV, Duerr A, Fitzgerald DW, Mogg R (2008). Efficacy assessment of a cell-mediated immunity HIV-1 vaccine (the Step Study): a double-blind, randomised, placebo-controlled, test-of-concept trial.. Lancet.

[3] Yang OO (2008). Aiming for successful vaccine-induced HIV-1-specific cytotoxic T lymphocytes.. Aids.

[4] Yang OO (2008). Retracing our STEP towards a successful CTL-based HIV-1 vaccine.. Vaccine.

[5] Bennett MS, Ng HL, Dagarag M, Ali A, Yang OO (2007). Epitope-dependent avidity thresholds for cytotoxic T-lymphocyte clearance of virus-infected cells.. J Virol.

[6] Bennett MS, Ng HL, Ali A, Yang OO (2008). Cross-clade detection of HIV-1-specific cytotoxic T lymphocytes does not reflect cross-clade antiviral activity.. J Infect Dis.

[7] Goulder PJ, Altfeld MA, Rosenberg ES, Nguyen T, Tang Y (2001). Substantial differences in specificity of HIV-specific cytotoxic T cells in acute and chronic HIV infection.. Journal of Experimental Medicine.

[8] Lichterfeld M, Yu XG, Cohen D, Addo MM, Malenfant J (2004). HIV-1 Nef is preferentially recognized by CD8 T cells in primary HIV-1 infection despite a relatively high degree of genetic diversity.. Aids.

[9] Borrow P, Lewicki H, Wei X, Horwitz MS, Peffer N (1997). Antiviral pressure exerted by HIV-1-specific cytotoxic T lymphocytes (CTLs) during primary infection demonstrated by rapid selection of CTL escape virus.. Nat Med.

[10] Jones NA, Wei X, Flower DR, Wong M, Michor F (2004). Determinants of human immunodeficiency virus type 1 escape from the primary CD8+ cytotoxic T lymphocyte response.. J Exp Med.

[11] Price DA, Goulder PJ, Klenerman P, Sewell AK, Easterbrook PJ (1997). Positive selection of HIV-1 cytotoxic T lymphocyte escape variants during primary infection.. Proc Natl Acad Sci U S A.

[12] Karlsson AC, Iversen AK, Chapman JM, de Oliviera T, Spotts G (2007). Sequential broadening of CTL responses in early HIV-1 infection is associated with viral escape.. PLoS ONE.

[13] Mattapallil JJ, Douek DC, Hill B, Nishimura Y, Martin M (2005). Massive infection and loss of memory CD4+ T cells in multiple tissues during acute SIV infection.. Nature.

[14] Mehandru S, Poles MA, Tenner-Racz K, Horowitz A, Hurley A (2004). Primary HIV-1 infection is associated with preferential depletion of CD4+ T lymphocytes from effector sites in the gastrointestinal tract.. J Exp Med.

[15] Veazey RS, Tham IC, Mansfield KG, DeMaria M, Forand AE (2000). Identifying the target cell in primary simian immunodeficiency virus (SIV) infection: highly activated memory CD4(+) T cells are rapidly eliminated in early SIV infection in vivo.. J Virol.

[16] Lichterfeld M, Yu XG, Mui SK, Williams KL, Trocha A (2007). Selective Depletion of High-Avidity Human Immunodeficiency Virus Type 1 (HIV-1)-Specific CD8+ T Cells after Early HIV-1 Infection.. J Virol.

[17] Ibarrondo FJ, Anton PA, Fuerst M, Ng HL, Wong JT (2005). Parallel human immunodeficiency virus type 1-specific CD8+ T-lymphocyte responses in blood and mucosa during chronic infection.. J Virol.

[18] Jones N, Agrawal D, Elrefaei M, Hanson A, Novitsky V (2003). Evaluation of antigen-specific responses using in vitro enriched T cells.. J Immunol Methods.

[19] Jacks T, Power MD, Masiarz FR, Luciw PA, Barr PJ (1988). Characterization of ribosomal frameshifting in HIV-1 gag-pol expression.. Nature.

[20] Yang OO, Kalams SA, Rosenzweig M, Trocha A, Jones N (1996). Efficient lysis of human immunodeficiency virus type 1-infected cells by cytotoxic T lymphocytes.. J Virol.

[21] Yang OO, Kalams SA, Trocha A, Cao H, Luster A (1997). Suppression of human immunodeficiency virus type 1 replication by CD8+ cells: evidence for HLA class I-restricted triggering of cytolytic and noncytolytic mechanisms.. J Virol.

[22] Korber B, Gaschen B, Yusim K, Thakallapally R, Kesmir C (2001). Evolutionary and immunological implications of contemporary HIV-1 variation.. Br Med Bull.

[23] Matthews PC, Leslie AJ, Katzourakis A, Crawford H, Payne R (2009). HLA footprints on human immunodeficiency virus type 1 are associated with interclade polymorphisms and intraclade phylogenetic clustering.. J Virol.

[24] Jin X, Bauer DE, Tuttleton SE, Lewin S, Gettie A (1999). Dramatic rise in plasma viremia after CD8(+) T cell depletion in simian immunodeficiency virus-infected macaques.. J Exp Med.

[25] Matano T, Shibata R, Siemon C, Connors M, Lane HC (1998). Administration of an anti-CD8 monoclonal antibody interferes with the clearance of chimeric simian/human immunodeficiency virus during primary infections of rhesus macaques.. J Virol.

[26] Schmitz JE, Kuroda MJ, Santra S, Sasseville VG, Simon MA (1999). Control of viremia in simian immunodeficiency virus infection by CD8+ lymphocytes.. Science.

[27] Momany C, Kovari LC, Prongay AJ, Keller W, Gitti RK (1996). Crystal structure of dimeric HIV-1 capsid protein.. Nat Struct Biol.

[28] Montero M, van Houten NE, Wang X, Scott JK (2008). The membrane-proximal external region of the human immunodeficiency virus type 1 envelope: dominant site of antibody neutralization and target for vaccine design.. Microbiol Mol Biol Rev.

[29] Lee CH, Saksela K, Mirza UA, Chait BT, Kuriyan J (1996). Crystal structure of the conserved core of HIV-1 Nef complexed with a Src family SH3 domain.. Cell.

[30] Kedl RM, Rees WA, Hildeman DA, Schaefer B, Mitchell T (2000). T cells compete for access to antigen-bearing antigen-presenting cells.. J Exp Med.

[31] Palmowski MJ, Choi EM, Hermans IF, Gilbert SC, Chen JL (2002). Competition between CTL narrows the immune response induced by prime-boost vaccination protocols.. J Immunol.

[32] Rodriguez F, Harkins S, Slifka MK, Whitton JL (2002). Immunodominance in virus-induced CD8(+) T-cell responses is dramatically modified by DNA immunization and is regulated by gamma interferon.. J Virol.

[33] Shapiro ED, Berg AT, Austrian R, Schroeder D, Parcells V (1991). The protective efficacy of polyvalent pneumococcal polysaccharide vaccine.. N Engl J Med.

[34] Reisinger KS, Block SL, Lazcano-Ponce E, Samakoses R, Esser MT (2007). Safety and persistent immunogenicity of a quadrivalent human papillomavirus types 6, 11, 16, 18 L1 virus-like particle vaccine in preadolescents and adolescents: a randomized controlled trial.. Pediatr Infect Dis J.

[35] Rolland M, Nickle DC, Mullins JI (2007). HIV-1 group M conserved elements vaccine.. PLoS Pathog.

[36] Letourneau S, Im EJ, Mashishi T, Brereton C, Bridgeman A (2007). Design and pre-clinical evaluation of a universal HIV-1 vaccine.. PLoS ONE.

[37] Kiepiela P, Ngumbela K, Thobakgale C, Ramduth D, Honeyborne I (2007). CD8+ T-cell responses to different HIV proteins have discordant associations with viral load.. Nat Med.

[38] Novitsky V, Gilbert P, Peter T, McLane MF, Gaolekwe S (2003). Association between virus-specific T-cell responses and plasma viral load in human immunodeficiency virus type 1 subtype C infection.. J Virol.

[39] Riviere Y, McChesney MB, Porrot F, Tanneau-Salvadori F, Sansonetti P (1995). Gag-specific cytotoxic responses to HIV type 1 are associated with a decreased risk of progression to AIDS-related complex or AIDS.. AIDS Res Hum Retroviruses.

[40] Rolland M, Heckerman D, Deng W, Rousseau CM, Coovadia H (2008). Broad and Gag-biased HIV-1 epitope repertoires are associated with lower viral loads.. PLoS ONE.

[41] Heckerman D, Frahm N, Pereyra F, Dubey S, Geraghty D Vaccine-induced targeting of epitopes associated with spontaneous control of HIV viral replication is associated with lower set-point viral loads in HIV-infected participants from the STEP trial; 2009;.

[42] Frahm N, Baker B, Brander C, Korber BTM, Brander C, Haynes B, Koup R, Moore JP (2008). Identification and Optimal Definition of HIV-Derived Cytotoxic T Lymphocyte (CTL) Epitopes for the Study of CTL Escape, Functional Avidity, and Viral Evolution.. HIV Molecular Immunology 2008.

